# Effect of solution treatment on the fatigue behavior of an as-forged Mg-Zn-Y-Zr alloy

**DOI:** 10.1038/srep23955

**Published:** 2016-04-01

**Authors:** S. D. Wang, D. K. Xu, B. J. Wang, E. H. Han, C. Dong

**Affiliations:** 1 Environmental Corrosion Center, Institute of Metal Research, Chinese Academy of Sciences, 62 Wencui Road, Shenyang 110016, China; 2 Laboratory of Materials Modification by Laser, Electron and Ion Beams, School of Materials Science and Engineering, Dalian University of Technology, Dalian 116024, China

## Abstract

Through investigating and comparing the fatigue behavior of an as-forged Mg-6.7Zn-1.3Y-0.6Zr (wt.%) alloy before and after solid solution treatment (T4) in laboratory air, the effect of T4 treatment on fatigue crack initiation was disclosed. S-N curves illustrated that the fatigue strength of as-forged samples was 110 MPa, whereas the fatigue strength of T4 samples was only 80 MPa. Observations to fracture surfaces demonstrated that for as-forged samples, fatigue crack initiation sites were covered with a layer of oxide film. However, due to the coarse grain structure and the dissolution of MgZn_2_ precipitates, the activation and accumulation of {10–12} twins in T4 samples were much easier, resulting in the preferential fatigue crack initiation at cracked twin boundaries (TBs). Surface characterization demonstrated that TB cracking was mainly ascribed to the incompatible plastic deformation in the twinned area and nearby α-Mg matrix.

Recently, extensive research work indicated that I-phase (Mg_3_Zn_6_Y, icosahedral quasicrystal structure, quasi-periodically ordered) strengthened Mg-Zn-Y-(Zr) alloys exhibit superior mechanical properties at both ambient and elevated temperatures^1^,^2^. Appropriate thermo-mechanical processing (such as hot rolling, hot extrusion or forging) can severely cleave I-phase network into dispersive segments in the α-Mg matrix, leading to a substantial improvement in mechanical strength^3^. Thus, I-phase strengthened Mg-Zn-Y-Zr alloys could be considered as material candidates for the applications in aerospace and automobile industries, where both high strength and lightweight are desired. Moreover, before new Mg alloys being approved to serve as engineering materials, in particular as heavy load-bearing components, it is essential to evaluate their fatigue behavior and deeply understand the associated failure mechanism^4^,^5^,^6^,^7^,^8^.

Previous studies revealed that the fatigue crack initiation of wrought Mg alloys was mainly ascribed to inclusions, cyclic slip deformation and twin boundary (TB) cracking^7^,^9^,^10^. A proper example can be seen in an as-forged-T5 Mg-Zn-Y-Zr alloy, where the fatigue strength at 10^7^ cycles was as low as 50 MPa given the initiation of fatigue cracks at subsurface or surface inclusions^7^. In addition, sample surface could host initiation of fatigue cracks due to cyclic slip deformation in as-rolled Mg alloy AZ31^9^. Owing to the poor corrosion resistance of an as-forged-T5 Mg-6.26Zn-0.66Y-0.78Zr alloy (wt.%), Xu *et al.* reported the crack initiation sites were covered with an oxide layer when fatigue samples were tested in ambient air with 40–60% relative humidity^8^. Recently, it was indicated that TBs could be preferential sites to generate fatigue cracks in extruded Mg alloy AZ31^10^. In the study of fatigue behavior of pure Mg, Xu *et al.* demonstrated that the activation and subsequent accumulation of twins occurred even at a low stress amplitude of 30 MPa^11^. Due to the incompatible plastic deformation occurred in the twinned area and the matrix, fatigue cracks preferentially initiated at the cracked TBs^11^. Additionally, for Mg alloys, the activation of deformation twinning increases with increasing grain size^12^,^13^,^14^. In the research about the compressive behavior of Mg alloy AZ91, Lahaie *et al.* reported that the deformation twinning was more likely to be activated in coarse grain-structured alloy (i.e. grain size of 15 μm) than that in the alloy with fine grain structure (i.e. grain size of 1 μm)^12^. Moreover, the deformation twinning at room temperature can hardly occur due to the strong suppression effect from precipitates^15^,^16^,^17^. Following this, it can be predicted that the activation and accumulation of twinning should be more probable in Mg alloys exhibiting a coarser grain structure and few precipitates. In previous work, Wang *et al.* reported that a solid solution treatment (T4) could simultaneously induce grain growth and dissolution of MgZn_2_ precipitates for an as-forged Mg-Zn-Y-Zr alloy^18^. Therefore, the fatigue crack initiation of the T4-treated Mg-Zn-Y-Zr alloys should be closely related to the activation and accumulation of twins. However, so far, few relevant work about the effect of solution treatment on fatigue strength degradation can be referred^6^. Moreover, it remains unclear whether a transition of fatigue crack initiation mechanism due to the T4 treatment exists.

Based on the above description, the present work aims to investigate the effect of microstructural changes derived from solution treatment on the fatigue behavior of an as-forged Mg-Zn-Y-Zr alloy. Additionally, the underlying fatigue crack initiation mechanism and possible transition for the alloy before and after T4 treatment will be discussed in detail.

## Results

### Three-dimensional X-ray computed tomography (XCT)

**Figure 1:**
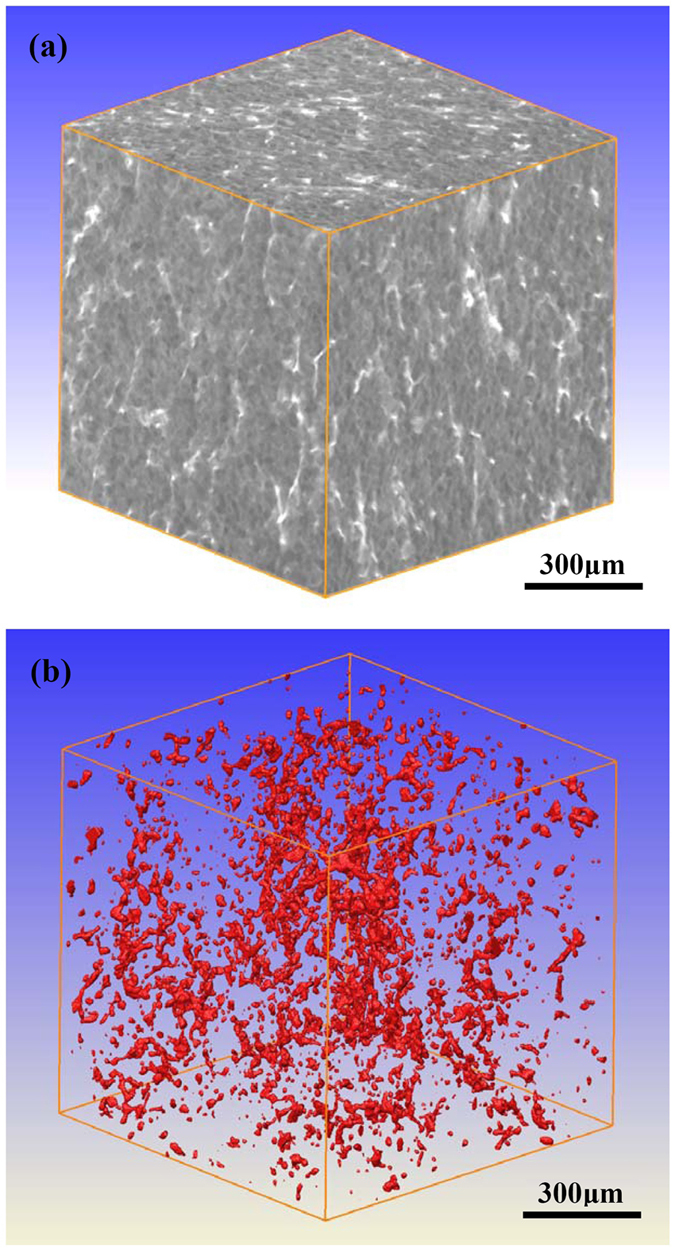
XCT results of the as-forged sample: (**a**) cuboid 1008 × 1008 × 1008 μm^3^ with three cross sections showing the Mg-Zn-Y phase particles; (**b**) 3D view of the segmented Mg-Zn-Y phase particles in the same cuboid (here, the α-Mg matrix is transparent).

Figure 1 shows the XCT result of the as-forged sample. It reveals that the three-dimensional XCT image is mainly composed of grey and white components (Fig. 1a). Previous work indicated that the main second phases in the as-forged Mg-6.7%Zn-1.3%Y-0.6%Zr alloy included coarse I-phase, a little of W-phase (Mg_3_Zn_3_Y_2_) and a high density of fine MgZn_2_ precipitates^18^. Among them, the formation of MgZn_2_ precipitates was due to the non-thermal holding during the hot forging process at elevated temperatures. Due to the particles with high mass density having strong absorption of the X-ray^19^, the bright component represents the Mg-Zn-Y phases (I-phase and W-phase) and the grey component should be the α-Mg matrix. Since the oxide inclusions or porosities with low mass density relative to the matrix have weak absorption of the X-ray, they should be dark if existed^19^. However, no dark areas could be observed within the resolution limit of XCT (Fig. 1a), indicating that the effective control of inclusions during the melting process and subsequent severe plastic deformation eliminated the possible existed non-metallic inclusions and porosities^8^. Even they could exist, their sizes should be smaller than 6 μm (supposing it requires at least two voxel points to construct an inclusion or a porosity). Moreover, no MgZn_2_ precipitates are observed because their size is less than 1 μm and much smaller than the voxel size used in XCT analysis^18^. Figure 1(b) shows 3D view of the segmented Mg-Zn-Y phase particles. It reveals that the diameter of I-phase/W-phase particles varies from 6–100 μm and they could aggregate at some particular sites.

### S-N curves

**Figure 2:**
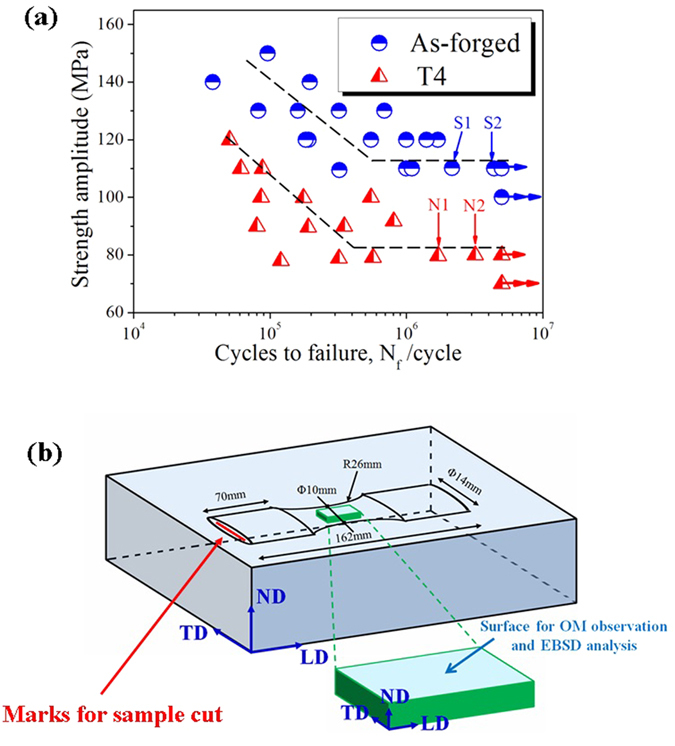
S-N curves for the as-forged and T4 treated samples that were cycled at a constant amplitude until failure or at least 5 × 10^6^ cycles were reached (whichever arrived first) (**a**). The dimensions and orientation of the fatigue samples and schematic to the surfaces of fatigue tested samples for OM observation and EBSD analysis (**b**). Orientations of ND, TD and LD in the samples were defined as normal, transverse and longitudinal directions of the plate, respectively. Mark for sample cut and surface for OM observation and EBSD analysis were also labeled.

Fatigue measurements of the as-forged and T4 treated samples were conducted and results were plotted as S-N curves (Fig. 2a). Regarding the as-forged samples, the fatigue strength was 110 MPa when 5 × 10^6^ cycles were employed, whereas the corresponding fatigue strength of T4 samples was as low as 80 MPa. In previous work, Ogarrevic and Stephens suggested that the fatigue ratio between fatigue strength (σ_−1_) and ultimate tensile strength (σ_b_) was 0.25–0.5 for wrought Mg alloys and higher ratios corresponded to higher strength alloys^20^. Since the ultimate tensile strength (σ_b_) values of the as-forged and T4 samples are 280 and 262 MPa^18^, their fatigue ratios (σ_−1_/σ_b_) at 5 × 10^6^ cycles are 0.39 and 0.31, respectively.

### Surface characterization of the samples subjected to fatigue test

**Figure 3:**
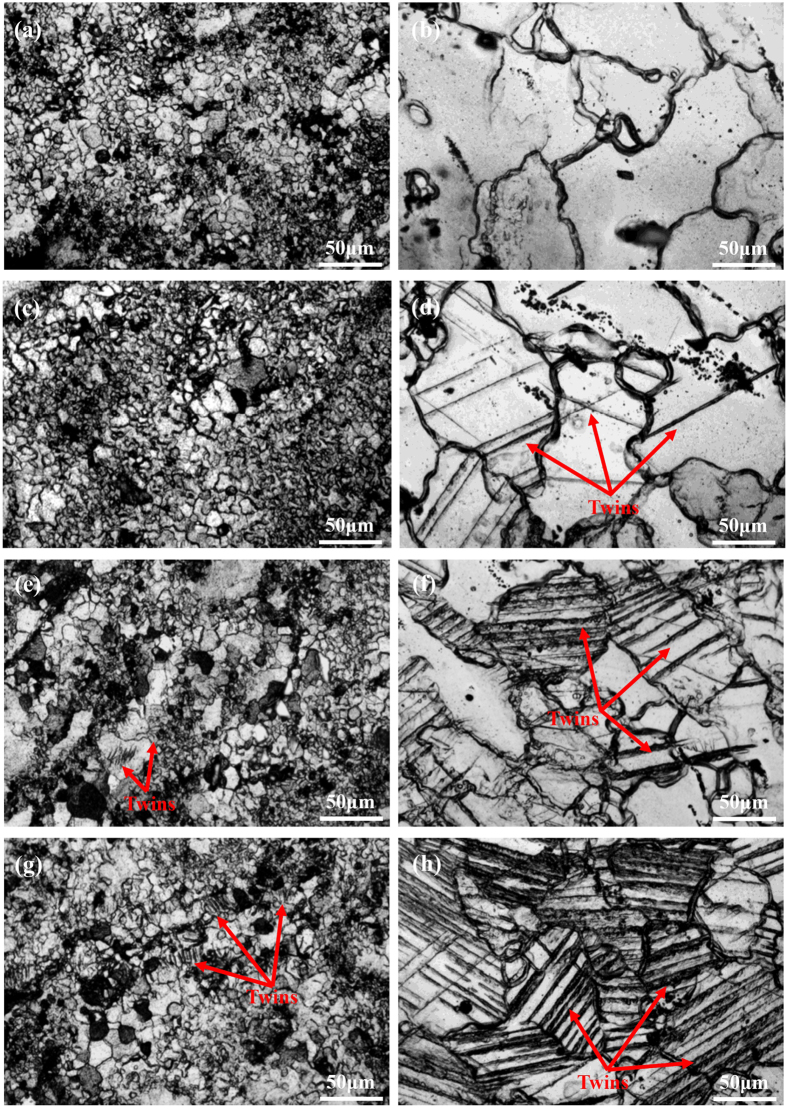
Optical microscopy observation to the as-forged and T4 treated samples subjected to fatigue testing at stress amplitudes of 100 MPa and 70 MPa respectively for: (**a,b**) 0, (**c,d**) 10^3^, (**e,f**) 10^5^ and (**g,h**) 5 × 10^6^ cycles. Images of (**a**,**c**,**e**,**g**) were taken on an as-forged sample surface; Images (**b**,**d**,**f**,**h**) were taken on a T4 treated sample surface.

To understand the initiation mechanism of fatigue cracks, microstructural alterations of the as-forged and T4 treated samples subjected to fatigue testing over a numbers of cycles were examined (Fig. 3). In terms of the as-forged samples, the twins were hardly activated during the cyclic loading at a stress amplitude of 100 MPa (Fig. 3a,c,e,g). On the contrary, after T4 treatment, the density of activated twins increased remarkably with loading cycles (Fig. 3b,d,f,h) even at a low stress amplitude of 70 MPa. After 5 × 10^6^ cycles, a high density of activated twins was observed and occurred nearly in all grains (Fig. 3h). Therefore, it demonstrates that twinning is reluctant to be activated in the as-forged samples, which consequently contributes little to the initiation of fatigue cracks. On the contrary, twinning can be triggered readily in the T4 treated samples and their subsequent accumulation may play a dominant role in inducing the fatigue crack intiation.

**Figure 4:**
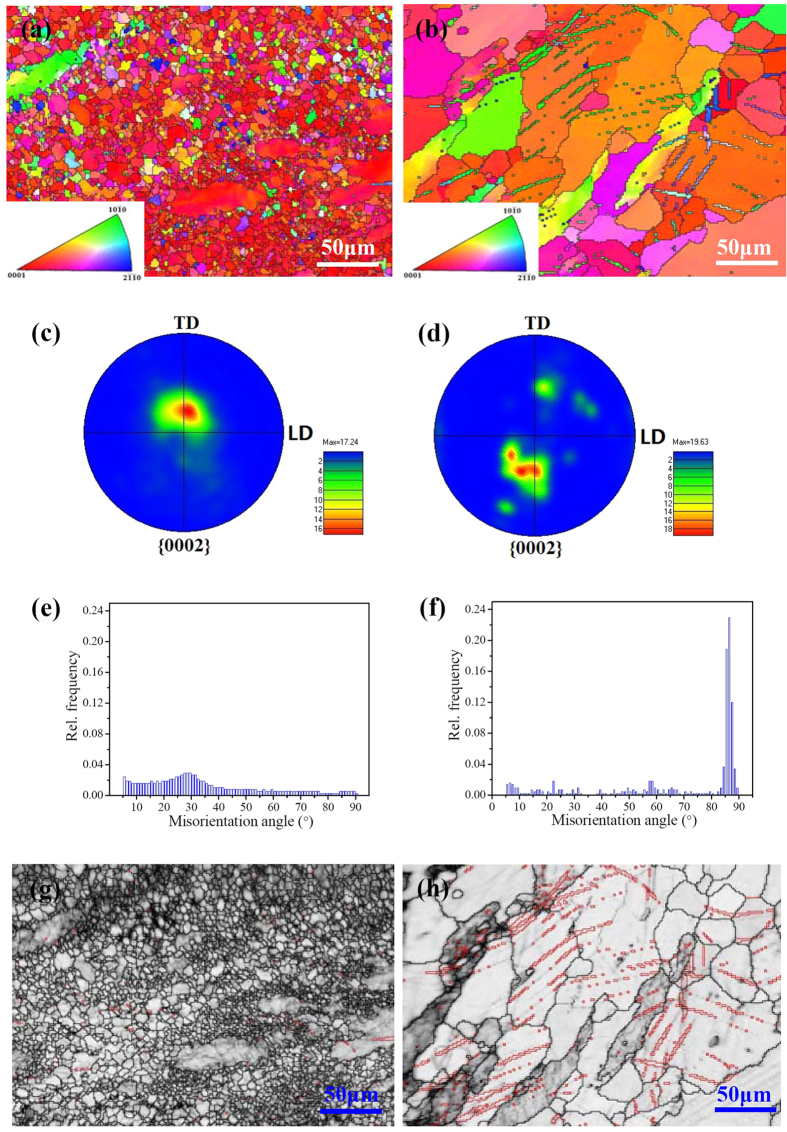
EBSD data of the surface of fatigue samples survived after 5 × 10^6^ cycles: (**a,c,e**,**g**) as-forged, 100 MPa; (**b,d,f**,**h**) T4, 70 MPa; (**a,b**) EBSD orientation maps to the grain structure of the as-forged and T4 samples; (**c,d**) {0002} pole figures of the as-forged and T4 samples; (**e,f**) misorientation angle distributions of the as-forged and T4 samples; (**g,h**) {10–12} twin boundaries (i.e. 86° < 11–20> ± 5°) were labeled in red in the as-forged and T4 samples. It is to be noted that the inverse pole figures reflecting the orientation relationship between the sample surfaces and crystallographic planes of grains were inserted in images (**a**,**b**).

The twinning modes of the as-forged and T4 treated samples subjected to fatigue testing up to 5 × 10^6^ cycles were determined by EBSD analysis (Fig. 4). The colors in the inverse pole figure maps correspond to the crystallographic axes of grains with the index digrams as insets. It reveals that for both samples, the orientations of {0002} basal plane in most grains were concentrated and parallel to the sample surface to a great degree, whereas only a few orientations of {10–10} and {11–20} prism planes were present. The {0002} pole figures of the as-forged and T4 samples (Fig. 4(c,d)) indicate that the c-axis of most grains in two samples were approximately aligned with the normal direction of the plate. This therefore confirms that surfaces of both samples were mainly composed of {0002} basal plane. To avoid the influence induced by the noise during EBSD measurement, the misorientation angle distributions in the range of 5°–95° for two samples were selected for plotting (Fig. 4e,f). It reveals that the maxima misorientation angle is approximately 28° for the as-forged sample, whereas that of the T4 treated sample is approximately 86°. Previous research indicated that the peaks corresponding to misorientation angles of 38°, 56° and 86° were attributed to the presence of {10–11}–{10–12} double twins, {10–11}<10–12> contraction twins and {10–12}<10–11> extension twins, respectively^21^,^22^,^23^,^24^. For T4 samples, the strong peak at 86° indicates the occurrence of a high density of {10–12}<10–11> twins. Moreover, {10–12} twin boundaries with misorientation angles and axes within 5° (i.e. 86° <11–20> ± 5°) were labeled for the as-forged and T4 treated samples (Fig. 4g,h). It can be seen that the density of {10–12} twins occurred in T4 samples was much higher than that of the as-forged samples, indicating that the activation and accumulation of twins underwent more readily in T4 samples.

**Figure 5:**
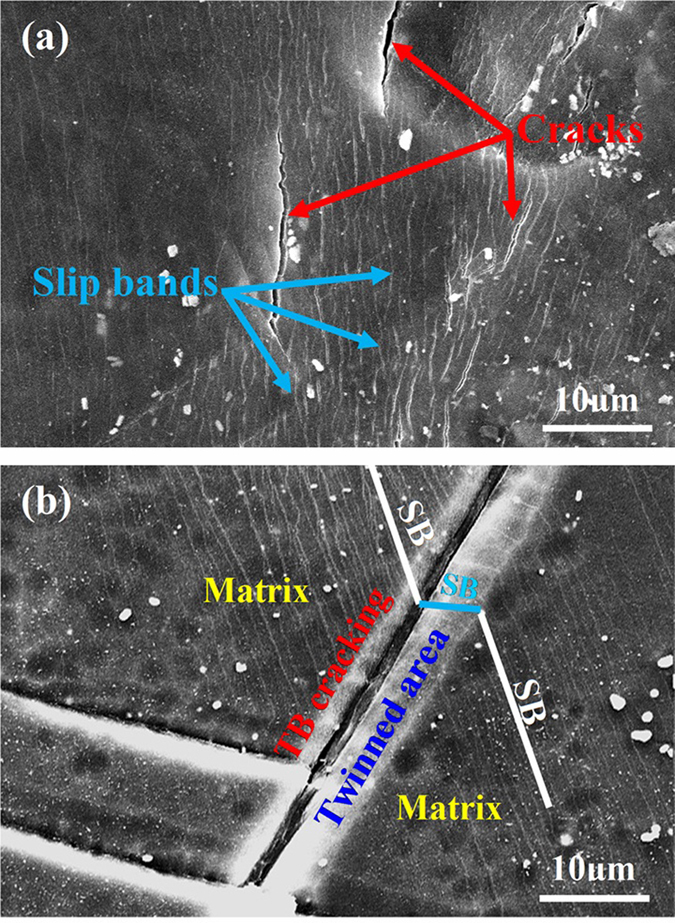
SEM observations to the surface of the fatigue samples survived after 5 × 10^6^ cycles: (**a**) as-forged, 100 MPa, showing microcracks initiate along slip bands; (**b**) T4, 70 MPa, showing microcracks initiate at twin boundaries. It is to be noted that “SB” and “TB” in image (**b**) denote slip band and twin boundary, respectively.

To elucidate the initiation mechanism of fatigue cracks, the gauge surfaces of as-forged and T4 treated samples were observed (Fig. 5). For the as-forged sample subjected to 5 × 10^6^ cycles at a stress amplitude of 100 MPa, intense slip bands emerged on the surface. Meanwhile, microcracks preferentially initiated along the intense slip bands (Fig. 5a). It was suggested that this kind of crack initiation is ascribed to cyclic slip deformation^25^. However, for the T4 sample subjected to 5 × 10^6^ cycles at a stress amplitude of 70 MPa, microcracks were mainly initiated at cracked TBs, though dense slip bands could form on the sample surface (Fig. 5b). Moreover, it demonstrates that at both sides of the cracked TBs, dense slip bands were observed in the twinned areas and the matrix (Fig. 5b).

### Fatigue crack initiation

**Figure 6:**
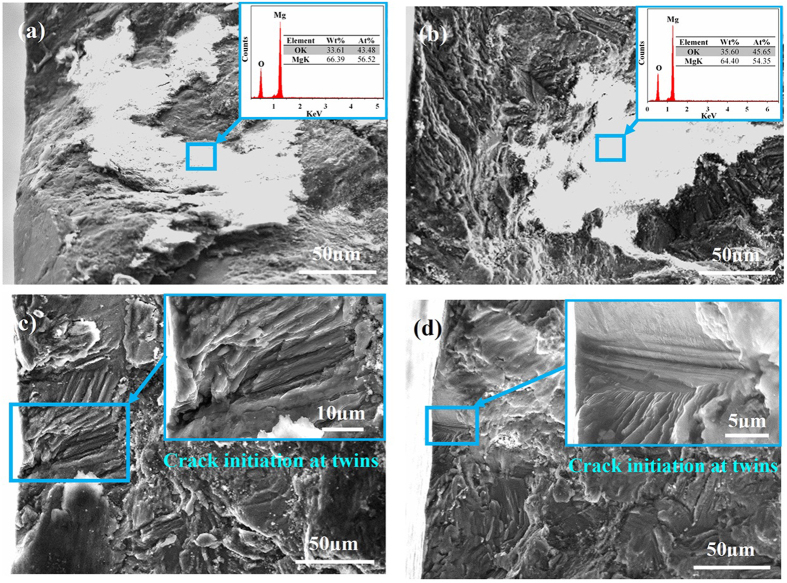
High-magnification observations of the fatigue fracture surface morphology of samples S1, S2, N1 and N2. Images (**a**,**b**) are secondary electron images of the oxide film located at the fatigue crack initiation regions of samples S1 and S2, respectively; Images (**c**,**d**) are secondary electron images of the crack initiation sites of samples N1 and N2, respectively. It should be noted that the inserts in images (**a**,**b**) are EDS results of the coresponding marked areas in the images; the inserts in images (**c**,**d**) are high-magnification images of the coresponding marked areas.

To discern the discrete initiation mechanisms of fatigue cracks occurred in as-forged and T4 samples, the fracture surfaces of four representative fatigue failed samples S1–S2 (as-forged condition) and N1–N2 (T4 condition) were studied (Fig. 2). SEM observations to fracture surfaces of samples demonstrate that for all conditions, fatigue cracks were preferentially initiated at sample surfaces, as shown in Fig. 6. It can be seen that for the as-forged samples, a layer of MgO (with a diameter of about 150 μm) covered on the crack initiation sites. Since no oxide inclusions or porosities were observed in the as-forged samples (Fig. 1), the MgO layer on the crack initiation sites should be formed during the fatigue process. However, the fatigue crack initiation sites of the T4 treated samples exhibited different characteristics. High-magnification images reveal that fatigue cracks were initiated at narrow flat planes and no oxide films were observed at crack initiation sites (Fig. 6c,d). Moreover, the microcracks were preferentially initiated at cracked TBs on the sample surface (Fig. 5b). Thus, the fatigue crack initiation at a narrow flat plane on the fracture surface was only ascribed to TB cracking.

## Discussion

It is well known that the fatigue lifetime of engineering materials during a high cyclic fatigue testing is mostly determined by crack initiation process^26^,^27^. In the research on fatigue behavior of high-strength carbon steels, Chapetti *et al.* proposed that the crack initiation process could take up more than 90% of the total fatigue lifetime^27^. It means that a sample will break up rapidly once fatigue crack is initiated. As such, this work focused on the study and analysis of crack initiation mechanism of Mg alloys. In general, {0001} <11–20> basal slip and {10–12} <10–11> twinning are two key plastic deformation mechanisms for Mg alloys at room temperature^28^. To understand their performance, the critical resolved shear stress (CRSS) for basal slip and twinning should be considered^29^. It has been widely accepted that at room temperature, CRSS value for the basal slip varies between 0.5–1 MPa, whereas the CRSS value for the {10–12} <10–11> twinning falls in a range of 4–10 MPa in pure Mg^29^,^30^,^31^,^32^. Following this, the activation of basal slip should be more likely than twinning during fatigue process. Moreover, with grain size decreasing, the formation of twin interfaces requires an extra driving force, which places more barriers for twinning^33^. Recently, Barnett *et al.* reported that a {10–12} tension twinning mode exhibits a stronger grain size effect than slip does^13^. Additionally, it was proposed that through regulating grain size down to a certain degree, the dominant deformation mode of Mg alloys at room temperature could transit from twinning to slip^13^. Meanwhile, Meyers *et al.* developed a constitutive approach and predicted through calculations that the deformation twinning at room temperature is prone to be activated in large grains (i.e. 100 μm) rather than in small grains (i.e. 3 μm) in hcp-structured metals^34^. Thus, it can be estimated that for the Mg alloys with finer grain structure, basal slip dominates the plastic deformation and the activation of twinning is restricted. Moreover, deformation twinning can be suppressed by precipitates in Mg alloys^15^,^16^,^17^. In the research of Mg alloy AZ91, Clark *et al.* suggested that deformation twinning at room temperature can hardly occur due to the strong suppression effect of the Mg_17_Al_12_ precipitates in monotonic loading test^16^. Similarly, Jain *et al.* reported that the presence of Mg_17_Al_12_ precipitates could significantly reduce the density of activated {10–12} tension twinning in tensile and compression tests^15^. Previous work indicated that after T4 treatment, the high density of MgZn_2_ precipitates were dissloved and the grain size was increased about 7.5 times^18^. Thus, the as-forged samples tend to perform cyclic slip, whilst T4 samples tend to perform cyclic slip and {10–12} <10–11> twinning during fatigue process, which is consistent with the microstructural changes of samples subjected to fatigue tests at various numbers of fatigue cycles (Figs 3, 4, 5).

Based on the microstructural changes of as-forged samples during fatigue process (Fig. 3), it reveals that the effect of twinning on fatigue crack initiation can be neglected. Meanwhile, slip bands can be widely activated (Fig. 5a). It has been reported that, during the deformation at room temperature, CRSS for prismatic slip was about 100 times higher than that for basal slip^35^,^36^. In fatigue testing, the loading amplitude is very small. Thus, only the {0001} <11–20> basal slip can be activated during the fatigue process. Since the relative humidity in air was 40–60% and the loading frequency was only 5 Hz, the time was adequate for O_2_/H_2_O molecules to adhere to the exposed slip step and cause the formation of oxide film during the tensile load of the former half cycle. When the load reversed in the latter half cycle, the adsorbed O_2_/H_2_O molecules and formed oxide film will be transferred along the slip band into the interior of the sample. Due to the inhibiting effect of the oxide film, it is a challenge for the reverse slip on the same slip plane and extra deformation on the slip plane will be created during subsequent fatigue cycles^8^. Based on the high irreversibility of cyclic slips^37^,^38^,^39^, extra deformation will lead to great stress concentration on the slip plane. Subsequently, cracks were preferentially initiated at the slip plane associated with a layer of oxide film (Fig. 6a,b).

For the T4 treated samples, both cyclic slip and deformation twinning could occur on the sample surface and no cracks were observed along slip bands. This reveals that the slip band cracking was suppressed due to a wide activation and accumulation of deformation twins. During fatigue process, cyclic slip took place in the twinned area and α-Mg matrix (Fig. 5b). Given the interactions between the environment and the fresh slip plane formed by intrusion/extrusions, an oxide layer can be formed on the slip plane. However, the cyclic slip deformation in the twinned area and nearby α-Mg matrix could cause severe deformation incompatibility at the TBs (Fig. 5b). With increased loading cycles, plastic deformation at TBs will gradually accumulate. When TBs can no longer accommodate the accumulated deformation incompatibility due to the complex deformation interactions, crack initiation will occur at the TBs^11^. Unlike the cyclic slip deformation, intrusion/extrusions could not occur at the TBs where accumulated deformation incompatibility is the main reason for stress concentrations. Thus, no oxide film has been observed at the cracked TBs (Fig. 6c,d). In comparison with as-forged samples, slip bands can hardly act as fatigue crack initiation sites of the T4 treated samples (Fig. 6). This implies that the local stress concentration at TBs was much higher than that at the slip bands, resulting in the preferential crack initiation at TBs.

In summary, the fatigue crack initiation for the as-forged sample was attributed to the irreversibility of cyclic slips induced by the retarding effect of oxide film on the reverse slip, whereas TB cracking is the dominant reason for fatigue crack initiation on fracture surfaces of T4 treated samples. When low stress amplitudes were employed, the stress concentrations at the slip bands and at the TBs were insufficient to create crack initiation or propagate any existing cracks. Therefore, the samples loaded at a low stress amplitude could survive after 5 × 10^6^ cycles (Fig. 2). In addition, it has been speculated that the local stress concentration at TBs is higher than that at slip bands. Compared with the as-forged sample, the cracks found in the T4 treated samples were much more likely to be initiated at a lower stress amplitude due to the severe activation of twinning and deformation incompatibility at TBs, which also explains why the fatigue strength of the T4 treated samples was remarkably lower than that of the as-forged samples.

## Methods

### Material preparation and treatments

The experimental material used for this investigation was an as-forged Mg-6.7%Zn-1.3%Y-0.6%Zr (in wt.%) plate with a thickness of 50 mm and a deformation ratio of 5. Sample pieces cut from the plate were annealed by a two-step treatment, i.e. holding at 300 °C for 1 h and then holding at 400 °C for 2 h (T4) in an air furnace, followed by quenching into room temperature water. The details for the proposed T4 treatment can be referred to the literature^18^.

### X-ray computed tomography (XCT)

To reveal the size and distribution of second phase particles and oxide inclusions in the investigated alloy, the advanced high-resolution three-dimensional X-ray tomography scanning (Xradia Versa XRM-500) was performed with an acceleration voltage of 80 kV and a 2048 × 2048 pixel array CCD detector equipped with a lens of 4×. The target used was made of tungsten. The voxel size used was (2.8723 μm)^3^ and the exposure time was 4 s for each of the 1600 projection images while the sample was rotated 360° along its vertical axis. The projection data were reconstructed to a volume dataset by means of a filtered back projection algorithm, and then, visualised and processed with the software Avizo Fire. A grey value was calculated for each position of the volume dataset (i.e. a volume element called voxel) in the scanned sample volume. The determined grey values correspond to the effective X-ray attenuation coefficient “μ” which is a function of the density and the atomic number of the elements within that voxel (x, y, z)^40^. Thus, areas with high mass density (i.e. Mg-Zn-Y phases) relative to the Mg matrix appear bright whereas the areas with low mass density (i.e. porosities or oxide inclusions containing Mg and O) should be dark in the reconstructed images. Further details on the XCT device can be found elsewhere^19^,^40^,^41^,^42^,^43^.

### Microstructural analysis

Microstructural evolution of the etched samples as a function of cycles of the fatigue process was examined through optical microscopy (OM), scanning electron microscopy (SEM; XL30-FEG-ESEM) equipped with energy-dispersive X-ray spectroscopy (EDS). Moreover, to characterize the twinning mode occurred in fatigue tested samples, electron backscatter diffraction (EBSD) analyses were carried out using a SEM (Hitachi S-3400N) equipped with an Oxford Instruments-HKL Channel 5 EBSD system at an accelerating voltage of 20 kV, a step size of 1.5 μm and a sample tilt angle of 70°. To maintain an identical direction for both OM observation and EBSD analysis, i.e. along the normal direction (ND) of the plate, one side of the fatigue samples was marked before testing (Fig. 2b).

### Fatigue testing

Fatigue samples with a minimum diameter of 10 mm and a round radius of 26 mm were cut from the plate (Fig. 2b). Before fatigue testing, samples were mechanically polished by silicon carbide papers and then buff-polished to 1 μm finish with ethanol lubricant. Then, fatigue tests were conducted on a rotating bending fatigue machine (PQ1-6 type, produced by Tianshui Hongshan Testing Machine Co., Ltd., China) at a frequency of 5 Hz (300 rpm (rotations per minute)) and a stress ratio of R = −1 in laboratory air (temperature 25–30 °C, relative humidity 40–60%). Samples were cycled at constant stress amplitudes until failure or until at least 5 × 10^6^ cycles were reached.

### Failure analysis

Surface and fracture characteristics of the fatigue tested samples were observed by SEM (XL30-FEG-ESEM) in both secondary electron imaging (SEI) and backscattered electron imaging (BEI) modes. EDS analysis was employed to determine the chemical composition of crack initiation sites on fracture surfaces.

## Additional Information

**How to cite this article**: Wang, S. D. *et al.* Effect of solution treatment on the fatigue behavior of an as-forged Mg-Zn-Y-Zr alloy. *Sci. Rep.* **6**, 23955; doi: 10.1038/srep23955 (2016).
